# Regulation of the Subjective Experience of Safety in Humor among Younger Schoolchildren

**DOI:** 10.1192/j.eurpsy.2025.1901

**Published:** 2025-08-26

**Authors:** A. Akhmetzyanova, T. Artemyeva, I. Artemyeva

**Affiliations:** 1 Department of Psychology and Pedagogy of Special Education, Kazan Federal University, Kazan; 2 Institute of Pedagogy and Psychology, Moscow State Pedagogical University, Moscow, Russian Federation

## Abstract

**Introduction:**

Laughter can be regarded as a distinct form of cathartic experience, arising from the violation of various well-established cultural norms. The balance between the emotions of laughter and fear depends on the extent and nature of the norm violation, as well as the specifics of its perception. The development of regulatory functions enables younger schoolchildren to comprehend increasingly complex forms of humor, which involve the transgression of cultural norms—ranging from behavioral violations to breaches of logical and linguistic norms.

**Objectives:**

To examine the specific features of humor perception and production by younger schoolchildren, as well as the potential for regulating their subjective experiences of danger and safety in situations involving norm violations in humor.

**Methods:**

The empirical study involved 360 younger schoolchildren (aged 7-10). The “Sometimes It Happens” (T. Artemyeva) method was used to study humor. The analysis of the schoolchildren’s responses was based on the following indicators: identification of rules (norms) in academic, extracurricular, and family interaction situations; the creation of humorous event scenarios; and the selection of event outcomes (adaptive or maladaptive humor).

**Results:**

The study revealed that the development of regulatory functions in younger schoolchildren allows them not to fear violating certain cultural norms or rules of social interaction in a joke. The positive correlation between maladaptive event outcomes and the identification of norms and rules by younger schoolchildren indicates the development of their regulatory function, mastery over their own cognitive processes, and their ability to interpret “dangerous” or “fear-inducing” scenarios as subjectively non-threatening, allowing them to emotionally distance themselves from such situations. Younger schoolchildren recognize the potential of humor in violating cultural norms, understanding that something in the event does not occur as it should according to the rule. In the child’s view, adherence to cultural norms guarantees a sense of safety in interactions with adults and peers.

**Conclusions:**

The findings of the study provide a foundation for the development of programs aimed at enhancing the regulatory functions of younger schoolchildren.

This paper has been supported by the Kazan Federal University Strategic Academic Leadership Program (PRIORITY-2030).

**Disclosure of Interest:**

None Declared

